# Multiple System Atrophy (Cerebellar Type) With Overlapping Progressive Muscular Atrophy Features and Genetic Erb-B2 Receptor Tyrosine Kinase 4 (ERBB4) Amyotrophic Lateral Sclerosis Variant: A Case Report

**DOI:** 10.7759/cureus.82509

**Published:** 2025-04-18

**Authors:** Enrique Lorenzo C Panganiban, Raymond L Rosales

**Affiliations:** 1 Section of Adult Neurology, Institute for Neurosciences, St. Luke's Medical Center, Quezon City, PHL

**Keywords:** amyotrophic lateral sclerosis, dysautonomia, erbb4 gene, lower motor neuron disease, multiple system atrophy

## Abstract

Multiple system atrophy (MSA) is a progressive disease with Parkinsonism, dysautonomia, and cerebellar symptoms wherein patients can present with a broad range of confusing and overlapping findings attributable to various neuroanatomical substrates. Although possible, weakness is an unusual primary complaint, warranting further work-up for another neurodegenerative disease. The involvement of the more central structures, such as the locus coeruleus, pontine micturition center, and the cerebellum, can explain the wide range of symptoms. While Onuf's nucleus contributes to the urinary symptoms, anterior horn cells can implicate a motor neuron disease. Taking the varied neuroanatomical substrates into consideration, patients can present with a plethora of dysregulated motor symptoms. The authors share the course of a patient with clinically established MSA-cerebellar type and lower motor neuron disease findings at par with progressive muscular atrophy (PMA), but tested positive for an ERBB4 gene mutation, which is linked to an amyotrophic lateral sclerosis (ALS) variant.

A 65-year-old Chinese female manifested with bilateral leg weakness and urinary incontinence. Over the next five years, she developed recurrent pre-syncopal attacks, asymmetric limb tremors, memory lapses, laughing fits, and a staccato-like voice. Medical management with anti-Parkinsonism drugs did not help her condition. Repeated annual non-contrast enhanced cranial magnetic resonance imaging (MRI) revealed gradual cerebellar atrophy, and an eventual prominent "hot-cross bun" sign. Because of episodes of orthostatic hypotension, with a systolic blood pressure as low as 50 mmHg, she gradually became bedridden with progressive arm weakness and sleep issues. These prompted her admission. Saccadic dysmetria and ataxic dysarthria aided in the diagnosis of MSA-cerebellar type, while motor neuron disease findings included tongue fasciculation, asymmetric leg atrophy, and polyminimyoclonus, suggestive of PMA. Neurophysiological studies confirmed this, while whole genome sequencing yielded an ERBB4 gene ALS variant of uncertain significance. She remained compliant with physical therapy during her admission. Although she was prescribed fludrocortisone for symptomatic relief and a two-week course of edaravone, she was discharged with minimal improvement and wheelchair-bound. However, the patient eventually expired two years afterward due to systemic complications. Although suspicion for a certain movement disorder can be initially made with physical examination, diagnostics can shed further light on the patient's pathology, exemplifying the uniqueness of this case report and how varying neurodegenerative movement disorders can coexist in a single patient.

## Introduction

As an alpha-synucleinopathy, characterized by the presence of glial cytoplasmic inclusions, multiple systemic atrophy (MSA) is an adult-onset, sporadic, relentlessly progressive neurodegenerative disease characterized by varying degrees of Parkinsonian symptoms, dysautonomia, and cerebellar involvement. It is caused by selective atrophy and neuronal loss of the striatonigral and olivopontocerebellar systems, giving rise to its MSA-Parkinsonian (MSA-P) and MSA-cerebellar (MSA-C) subtypes [1,2]. Though the presenting hallmark of patients with MSA is recurrent syncope, as a result of orthostatic hypotension, a percentage of them may present with urinary incontinence as an initial complaint. However, there have been reports that MSA patients can also present with polyminimyclonus, rapid progression to wheelchair confinement, cognitive impairment, hallucinations, and even pathological laughter or crying [1,3].

On the other hand, while weakness can present in patients with Parkinsonism, it is not a common initial manifestation. There could be a possibility of an overlapping motor neuron pathology. Although differentiating between upper motor neuron versus lower motor neuron diseases can aid in the diagnosis, familial and sporadic types of MNDs can further narrow the differentials. Interestingly, the spine is involved in patients with MSA since the Onuf's nucleus, located in the sacral region, lies close to the somatic motor neurons within the anterior horn of the spinal cord, which can explain some symptoms of motor neuron diseases [4].

Given the multiple presentations, intracranial imaging and neurophysiological studies can confirm the diagnosis in such cases. However, the signs and symptoms can still be explained by certain neuroanatomic substrates such as the locus coeruleus. Genetic predisposition plays a significant role in symptom development, and prudent investigation of these defects should always be offered to the patient. Hence, the authors share how multiple neurodegenerative movement disorders can coexist in a single patient, specifically, clinically established MSA-C and lower motor neuron disease findings at par with progressive muscular atrophy (PMA), but tested positive for an ERBB4 gene, a heterozygous variant of uncertain significance, associated with the autosomal dominant amyotrophic lateral sclerosis (ALS).

## Case presentation

The case presented is a 65-year-old Chinese individual, ambulatory, with no history of trauma, but experienced bilateral gradual lower extremity weakness and gait imbalance, without any sensory deficits, which she had attributed to her low back pain from her previous spinal surgery five years prior to admission. She was able to perform her activities of daily living, but with increasing effort to lift her legs and developing slowness in gait. Three months later, she was admitted to a different hospital for urinary tract infection (UTI), urinary incontinence, and neurologic assessment. At the time, she presented with bilateral leg weakness, without sensory deficits, and an intact anal sphincteric reflex. A faint vertical, linear T2 hyperintensity within the pons, and cerebro-cerebellar atrophy was seen on non-contrast enhanced cranial MRI, while the first set of neurophysiological studies for her leg weakness yielded inconclusive results. She minimally improved on carbidopa-levodopa and did not return to her baseline.

The interim showed progressive weakness and more frequent urinary incontinence. Follow-up diagnostics showed a more distinct pontine linear T2 hyperintensity and further cerebellar atrophy on another MRI scan. During the pandemic, she was lost to follow-up and lived alone. She gradually deteriorated, requiring a walker and a diaper. Her voice became weak, poorly modulated, and staccato-like. She became irritable, bored, and disinterested in her family, which they had thought was due to the COVID lockdown.

**Figure 1:**
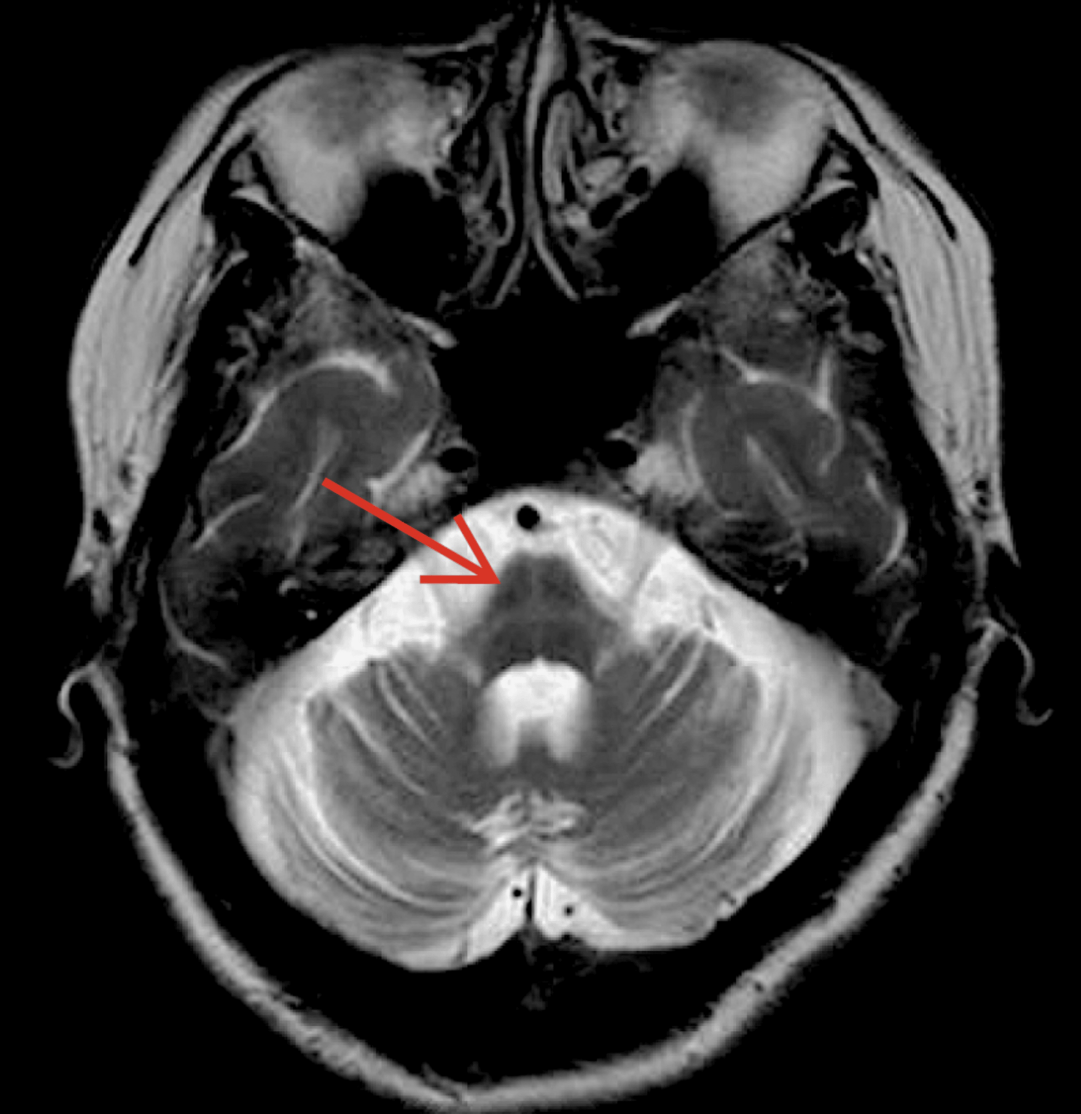
Cruciform pontine hyperintensity ("hot-cross bun" sign) seen in patients with MSA on cranial MRI (axial view) MSA - multiple system atrophy; MRI - magnetic resonance imaging

**Figure 2:**
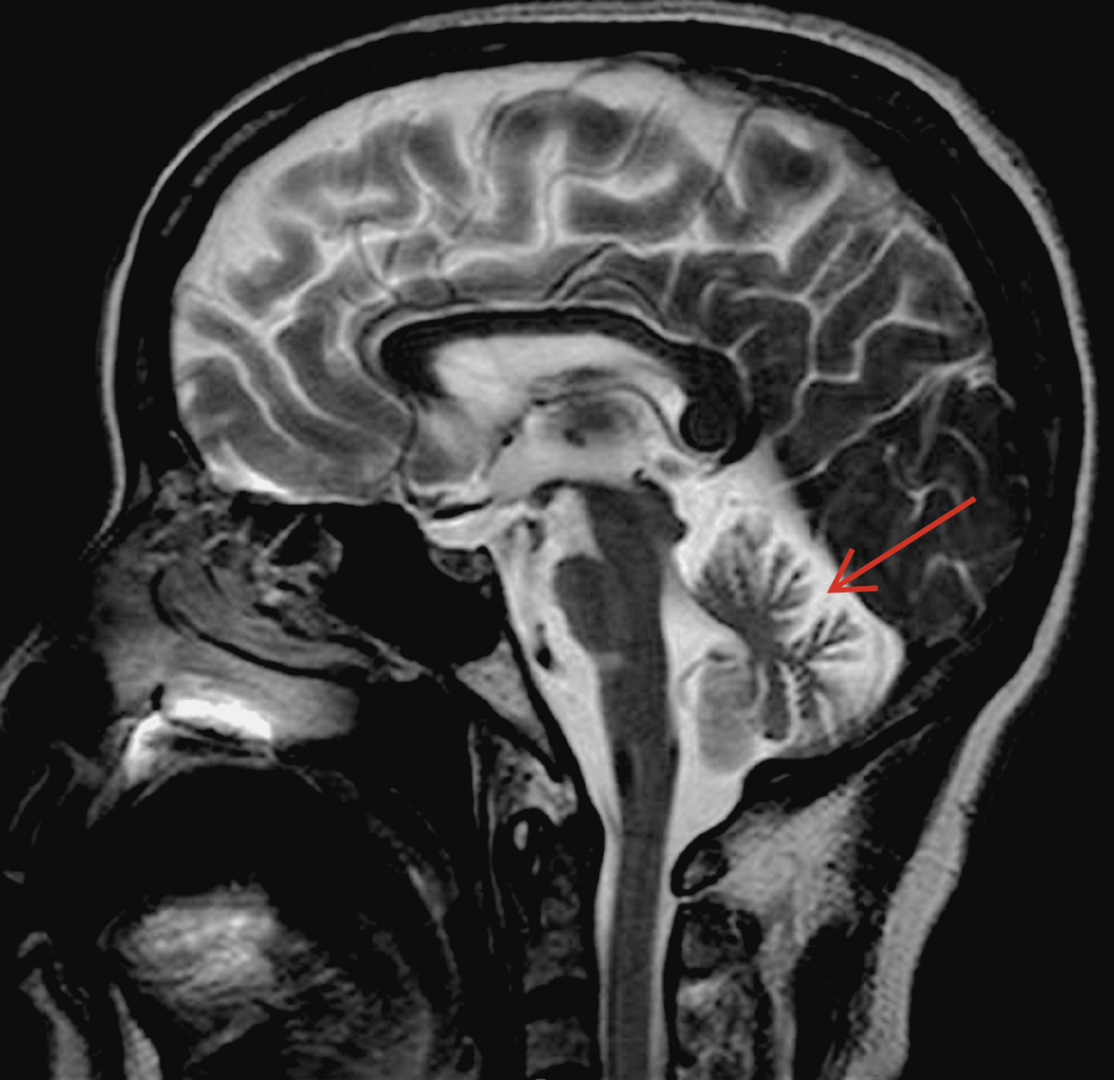
Marked cerebellar atrophy on cranial MRI (sagittal view)

Two years prior to admission, she was admitted for an episode of fall. A "hot-cross bun" sign within the pons was definitively seen on cranial MRI, and medications no longer improved her condition (Figures 1, 2). Aside from her recurrent UTI, she constantly experienced pre-syncopal episodes with orthostatic hypotension and became wheelchair-bound with asymmetric limb tremors. She was eventually managed as a case of MSA-C with features of PMA and was able to ambulate with assistance again after rehabilitation therapy. However, over the next year, she worsened again, now bed-bound with increasing memory lapses, misplaced laughing fits during conversations, beginning rapid eye movement (REM) sleep changes, described as acting out her dreams instead of the usual atonic state of sleep, complex visual hallucinations, daily pre-syncopal episodes during transfers, uncontrollable involuntary small amplitude, repetitive, non-rhythmic twitching of the hands and feet while awake and asleep, lasting for a few seconds, and gradual arm weakness. She was readmitted for treatment.

Physical examination elicited emotional lability, bilateral horizontal gaze nystagmus, saccadic dysmetria, ataxic dysarthria, intention and resting tremors, rigid arms, proximal muscle weakness, atrophy of the left gastrocnemius more than its contralateral counterpart, polyminimyoclonus, tongue fasciculations, areflexia, and hand dystonia. A repeat of neurophysiological testing (Table 1) revealed findings for a PMA. The whole exome sequencing yielded an ERBB4 gene mutation (p. Arg979Gln), which was classified as a heterozygous variant of uncertain significance associated with the autosomal dominant form of ALS-19. However, family history had denied any relative with similar symptoms. For two weeks, she was treated with fudrocortisone (100 mcg per tablet) once daily, improving her orthostatic hypotension, which was not documented by the conventional definition, and daily coenzyme Q10 tablets and intravenous edaravone, barely aiding her weakness. Furthermore, she was treated again for her urinary tract infection and underwent rehabilitation therapy twice a day, thus improving her leg strength, but she was still discharged wheelchair-bound. Two years afterward, she had succumbed to systemic complications.

**Table 1:** Electromyography report (St. Luke's Medical Center, Quezon City) CRD - complex repetitive discharges; PSW - positive sharp waves

Muscles	Spontaneous activity				
	Insertional activity	Fibrillations	Fasciculations	PSW	Motor unit potentials
Right tibialis anterior	Normal	-	-	-	6 Hz tremor, reduced recruitment pattern
Left tibialis anterior	Increased	-	-	++	Long duration, reduced recruitment pattern
Right triceps	Increased	-	-	+	Long duration, reduced recruitment pattern
Right first dorsal interosseous	Increased with CRD	+	+	++	Long duration, reduced recruitment pattern
Genioglossus	Increased	-	+	-	Long duration, reduced recruitment pattern

## Discussion

The case exemplifies how movement disorders can overlap with and even mask diseases of the motor neurons. The documented cerebellar symptoms were clearly seen in the progressive atrophy of this structure. However, the underlying neuroanatomic substrates attributed to blood pressure and urinary incontinence can be localized to numerous supraspinal structures involving the central autonomic network, such as the locus coeruleus within the pons. This provides much of the brain's neuromodulator, norepinephrine, which contributes to wakefulness, behavioral flexibility, memory, and cognition [5]. Historically, the "hot-cross bun" sign or the cruciform pontine hyperintensity was thought to be a pathognomonic sign specific for MSA. Although it does implicate structures that contributed to the patient's gradual deterioration and multiple symptoms, it has long since been refuted that this is the only pathology it is linked to and can be seen in other conditions [6]. Nonetheless, this radiologic finding increased the index of suspicion for MSA-C as the culprit of the patient's presentation.

However, the central location of these structures will not fully explain the lower motor neuron findings, such as the proximal asymmetric weakness, atrophy, and areflexia, warranting the search for another underlying pathology, which was a possible PMA despite the inconclusive findings on the first set of neurophysiological studies. Also essential to urinary bladder control is the sacral region of the spinal cord, where Onuf's nucleus is located. This structure may be involved in MSA; however, it is the adjacent motor neurons within the anterior horn of the lumbosacral spinal cord that can justify the weakness [4]. Given that the patient presented with Parkinsonian symptoms, dementia-like symptoms, and ALS, a corticostriatospinal degeneration (Muro disease) was considered, like the Japanese studied in the Kii-peninsula, but was no longer considered given the prominent dysautonomia in the case [7]. Despite the limited effective management in preventing both MSA and ALS, symptomatic treatment still offers some clinical response. In theory, supplying the decreased coenzyme Q10 levels should improve the mitochondrial dysfunction and vulnerability to oxidative stress in patients with MSA [8]. While edaravone attempts to increase mitochondrial membrane potential, and ATP levels, and reduce reactive oxygen species [9-10]. 

Though PMA was considered, it was an ALS variant involving the ERBB4 gene that the results indicated toward. The ErbB signaling pathway plays a key role in the development, maintenance, and repair of motor neurons within both the central and peripheral nervous systems. Part of the epidermal growth factor family of extracellular ligands, neuregulins bind to the ErbB family of receptor tyrosine kinases, whose downstream effects contribute to diverse neuronal function such as cell differentiation, recovery after nerve injury, synaptic and neuromuscular junction development, maturation and myelination of Schwann cells, and regulation of neurotransmitter release, especially the ErbB4 receptor family [11]. A Japanese study performed whole exome sequencing of the ErbB4 protein and documented ALS symptoms in patients with a genetic mutation (p. Arg927Gln) within the tyrosine kinase domain, which mediates the function of the protein [12]. Likewise, three possibly pathologic variants were also documented in a Chinese cohort (p. Arg106His, p. Gln164Pro, and p. Val212Leu) that did not carry common pathogenic mutations related to ALS such as SOD1, TARDBP, FUS, and C9ORF72. These variants are located upstream of the tyrosine kinase encoding region of the gene [12-13]. In its supplementary data, the same chromosomal DNA mutation (p. Arg979Gln) was documented in 3 of its 1812 controls who did not manifest with any neurological symptoms at the time of onset of the study, and none in the 448 patients who had neurologic symptoms [13]. This offers, then, the possibility that this variant, though still documented as of uncertain significance, can offer insight into the pathogenicity of ALS in patients with movement disorders, and is worth studying. Its implication may also contribute to the rapidity of disease development or even a predominant motor neuron disease presentation in patients with Parkinsonism. 

## Conclusions

Despite the great lengths clinical and genetic research has gone, a diagnostic dilemma still exists in patients with movement disorders. Diagnostic modalities can still bolster the primary impression despite both clinical acumen and exhaustive physical examination. In this patient, an index of suspicion guided clinicians to a diagnosis, but required other modalities to further confirm that both central and peripheral neurodegenerative diseases coexisted in this patient. This emphasizes the armamentarium of means a neurologist can use in patient care, from one's own senses to ancillary diagnostics. Furthermore, this case shows the difficulties that can be encountered in treating neurodegenerative movement disorders. 

## References

[1] Barrington CJ, Burruano M, Carney C (2021). Addressing the role of edaravone in the management of amyotrophic lateral sclerosis and gaps in care and access: expert panel recommendations. Am J Manag Care.

[2] Cha SJ, Kim K (2022). Effects of the edaravone, a drug approved for the treatment of amyotrophic lateral sclerosis, on mitochondrial function and neuroprotection. Antioxidants (Basel).

[3] Fanciulli A, Stankovic I, Krismer F, Seppi K, Levin J, Wenning GK (2019). Multiple system atrophy. Int Rev Neurobiol.

[4] Kuzuhara S, Kokubo Y (2005). Atypical parkinsonism of Japan: amyotrophic lateral sclerosis-parkinsonism-dementia complex of the Kii peninsula of Japan (Muro disease): an update. Mov Disord.

[5] Miki Y, Foti SC, Asi YT, Tsushima E, Quinn N, Ling H, Holton JL (2019). Improving diagnostic accuracy of multiple system atrophy: a clinicopathological study. Brain.

[6] Mitsui J, Tsuji S (2023). Coenzyme Q10 in multiple system atrophy. Trials for Cerebellar Ataxias. Contemporary Clinical Neuroscience.

[7] Neupane P, Thada PK, Singh P (2023). Investigating edaravone use for management of amyotrophic lateral sclerosis (ALS): a narrative review. Cureus.

[8] Ou GY, Lin WW, Zhao WJ (2021). Neuregulins in neurodegenerative diseases. Front Aging Neurosci.

[9] Prasad S, Rossi M (2022). The hot cross bun sign: a journey across etiologies. Mov Disord Clin Pract.

[10] Schellino R, Boido M, Vercelli A (2020). The dual nature of Onuf's nucleus: neuroanatomical features and peculiarities, in health and disease. Front Neuroanat.

[11] Takahashi Y, Fukuda Y, Yoshimura J (2013). ERBB4 mutations that disrupt the neuregulin-ErbB4 pathway cause amyotrophic lateral sclerosis type 19. Am J Hum Genet.

[12] Wang F, Liu X, He J, Zhang N, Chen L, Tang L, Fan D (2022). Analysis of ErbB4 variants in amyotrophic lateral sclerosis within a Chinese cohort. Front Neurol.

[13] Watanabe H, Shima S, Mizutani Y, Ueda A, Ito M (2023). Multiple system atrophy: advances in diagnosis and therapy. J Mov Disord.

